# Valorization of Riceberry Broken Rice and Soybean Meal for Optimized Production of Multifunctional Exopolysaccharide by *Bacillus tequilensis* PS21 with Potent Bioactivities Using Response Surface Methodology

**DOI:** 10.3390/polym17152029

**Published:** 2025-07-25

**Authors:** Thipphiya Karirat, Worachot Saengha, Nantaporn Sutthi, Pheeraya Chottanom, Sirirat Deeseenthum, Nyuk Ling Ma, Vijitra Luang-In

**Affiliations:** 1 Natural Antioxidant Innovation Research Unit, Department of Biotechnology, Faculty of Technology, Mahasarakham University, Maha Sarakham 41150, Thailand; thipphiya.k@gmail.com (T.K.); worachot207@gmail.com (W.S.); sirirat.d@msu.ac.th (S.D.); 2 Department of Agricultural Technology, Faculty of Technology, Mahasarakham University, Maha Sarakham 44150, Thailand; nantaporn.s@msu.ac.th; 3 Applied Animal and Aquatic Sciences Research Unit, Division of Fisheries, Faculty of Technology, Mahasarakham University, Maha Sarakham 44150, Thailand; 4 Department of Food Technology and Nutrition, Faculty of Technology, Mahasarakham University, Maha Sarakham 44150, Thailand; pheeraya.c@msu.ac.th; 5 Bioses Research Interest Group (BIOSES), Faculty of Science and Marine Environment, Universiti Malaysia Terengganu, Kuala Nerus 21030, Malaysia; nyukling@umt.edu.my

**Keywords:** agro-biowaste, anticancer, antimicrobial activity, antioxidant, tyrosinase, collagen

## Abstract

This study explores the valorization of agro-industrial by-products—riceberry broken rice (RBR) and soybean meal (SBM)—as cost-effective substrates for enhancing exopolysaccharide (EPS) production by *Bacillus tequilensis* PS21. Eight *Bacillus* strains were screened, and *B. tequilensis* PS21 demonstrated the highest EPS yield (2.54 g/100 mL DW). The EPS displayed a strong antioxidant capacity with 65.5% DPPH and 80.5% hydroxyl radical scavenging, and a FRAP value of 6.51 mg Fe^2+^/g DW. Antimicrobial testing showed inhibition zones up to 10.07 mm against *Streptococcus agalactiae* and 7.83 mm against *Staphylococcus aureus*. Optimization using central composite design (CCD) and the response surface methodology (RSM) revealed the best production at 5% (*w*/*v*) RBR, 3% (*w*/*v*) SBM, pH 6.66, and 39.51 °C, yielding 39.82 g/L EPS. This EPS is a moderate-molecular-weight (11,282 Da) homopolysaccharide with glucose monomers. X-ray diffraction (XRD) showed an amorphous pattern, favorable for solubility in biological applications. Thermogravimetric analysis (TGA) demonstrated thermal stability up to ~250 °C, supporting its suitability for high-temperature processing. EPS also exhibited anticancer activity with IC_50_ values of 226.60 µg/mL (MCF-7) and 224.30 µg/mL (HeLa) at 72 h, reduced colony formation, inhibited cell migration, and demonstrated anti-tyrosinase, anti-collagenase, and anti-elastase effects. This study demonstrates the successful valorization of agro-industrial by-products—RBR and SBM—for the high-yield production of multifunctional EPS with potent antioxidant, antimicrobial, and anticancer properties. The findings highlight the sustainable potential of these low-cost substrates in supporting the development of green and value-added bioproducts, with promising utilizations across the food, pharmaceutical, and cosmetic sectors.

## 1. Introduction

Several microbes release low- and high-molecular-weight polymers called exopolysaccharides (EPSs) into the environment. According to Abdel-Wahab et al. (2022), Nguyen et al. (2023), and Enrique et al. (2024) [1,2,3], these biomolecules have a wide range of biological activities, such as antibacterial, anti-inflammatory, immune-modulating, and therapeutic properties such as anticancer and wound healing effects. As a result, they are highly valuable for use in the food, pharmaceutical, cosmetic, and environmental industries.

*Bacillus* spp. have a number of desirable characteristics, including being a generally recognized as safe (GRAS) organism, being able to grow in a variety of environments, and secreting extracellular biopolymers that have bioactive properties [4,5]. Notably, EPSs from *Bacillus subtilis*, *Bacillus licheniformis*, and *Bacillus velezensis* have been reported to exhibit strong antioxidant and cytotoxic properties, targeting various human cancer cell lines, while also demonstrating promising use in cosmetic formulations as anti-aging and skin-whitening agents [4,6,7].

EPSs constitute a rapidly growing segment of the biopolymers industry. In 2021, the global polysaccharides market was estimated at USD 14.04 billion, with projections suggesting an increase to USD 21.41 billion by 2030, reflecting significant growth in demand and diverse applications [8]. For example, the xanthan gum market, a leading EPS, was valued at approximately USD 1 billion in 2019 and is expected to reach USD 1.5 billion by 2027. Similarly, the bacterial cellulose market, valued at USD 250 million in 2017, is anticipated to grow to USD 680 million by 2025. The pullulan market is also on an upward trajectory, with forecasts predicting growth from USD 65 million in 2022 to USD 89 million by 2030. Furthermore, the global dextran market, valued at USD 203.9 million in 2021, is projected to rise to USD 284.5 million by 2028 [9,10]. However, the large-scale production of EPSs is limited by the high costs associated with refined substrates and synthetic media [11]. A study comparing synthetic versus low-cost media using corn steep liquor and sugarcane molasses as substrates found that using alternative (non-refined) substrates can reduce nutrient costs by over 30-fold per kg of EPS produced compared with conventional synthetic media. This underscores how expensive refined/synthetic media are in driving total production expenses in industrial EPS fermentation, posing a significant barrier for EPS commercialization [11]. To address this, the biotechnological community has shifted focus toward the use of agricultural and food processing by-products, which are rich in the nitrogen and carbon necessary for microbial proliferation and metabolite biosynthesis. Examples include molasses, cassava pulp, fruit peels, and rice by-products [12,13,14,15,16]. These substrates not only reduce production costs, but also promote environmental sustainability by minimizing the organic waste and associated pollution risks.

Riceberry (*Oryza sativa* L.), a pigmented Thai rice variety, is primarily cultivated for its nutritional and functional food properties. During milling and processing, a significant proportion of broken rice is generated as a by-product [17]. Riceberry broken rice (RBR) is an appropriate carbon source for microbial fermentation due to its substantial quantity of fermentable carbohydrates (~75% in this work). Similarly, soybean meal (SBM), a protein-rich waste product of soybean oil processing, functions as a cost-effective nitrogen source [18]. Despite progress in this area, limited research has explored the combined use of RBR and SBM—two abundant agro-industrial by-products in Southeast Asia—as co-substrates for EPS production. Their co-utilization presents a novel approach to developing low-cost fermentation media for EPS production, yet their combined effects on microbial EPS yield and functionality remain underexplored. Moreover, few studies have systematically optimized the fermentation conditions for such substrates to maximize EPS yield and bioactivity while characterizing the resulting biopolymers comprehensively. This gap limits the ability to evaluate their full industrial potential.

Furthermore, EPSs derived from such agro-industrial by-products hold significant application value. It can serve as a bioactive ingredient in functional foods for health promotion (e.g., antioxidant and prebiotic effects), as a natural thickener or stabilizer in cosmetics, and as a pharmaceutical excipient with potential immunomodulatory or anticancer properties [1,3]. Such multifunctional applications align with the bio-circular-green (BCG) economic model and support the sustainable development goals (SDGs) by transforming agricultural waste into high-value bioproducts.

Despite the known potential of *Bacillus* spp. for EPS production, few studies have explored the co-utilization of RBR and SBM as fermentation substrates. This study addresses this gap by optimizing the culture conditions for EPS production using *Bacillus tequilensis* PS21, previously isolated from Thai milk kefir [19], using a response surface methodology (RSM). The statistical design considered the effects of four independent variables—RBR concentration, SBM concentration, initial pH, and temperature—on EPS yield.

Recent studies have highlighted the role of statistical modeling tools such as RSM in enhancing EPS production by fine-tuning process variables. For instance, Asgher et al. (2020) [4] employed a central composite design (CCD) to optimize EPS biosynthesis using B. licheniformis MS3 using wheat bran extract, achieving yields up to 3.4 g/L. Sathishkumar et al. (2021) [20] optimized EPS production using *B. subtilis* MKU SERB2, demonstrating the scalability of marine isolates under controlled fermentation. Our study builds upon these findings by integrating agro-waste valorization with statistical process control, thereby advancing sustainable EPS production models. In addition to process optimization, this study provides a comprehensive characterization of the EPS produced using *B. tequilensis* PS21 under optimized conditions. Structural analyses using scanning electron microscopy (SEM), nuclear magnetic resonance (NMR), Fourier-transform infrared spectroscopy (FTIR), gel permeation chromatography (GPC), X-ray diffraction (XRD), and thermogravimetric analysis (TGA) were performed to elucidate the morphology, molecular weight distribution, chemical composition, and thermal stability of the polymers. These features are important for determining whether the EPS is suitable for use in manufacturing, especially in industries such as medicine and cosmetics where maintaining molecular integrity during processing is paramount [3,21].

The biofunctional potential of the EPS was further assessed through antioxidant, antimicrobial, and anticancer assays. A high antioxidant capacity, determined by 2,2-diphenyl-1-picrylhydrazyl (DPPH) and hydroxyl radical scavenging, ferric reducing antioxidant power (FRAP), and total phenolic (TPC) and flavonoid content (TFC), suggests the presence of redox-active moieties within the EPS matrix [1,22]. This action lends credence to the concept that it could be useful in formulations that target oxidative stress-induced cellular damage [1,22]. The bacterial EPS additionally displayed notable antimicrobial effects against *Streptococcus agalactiae* and *Staphylococcus aureus*, highlighting its potential as a natural therapeutic agent [23]. Similar effects have been observed in EPS derived from *B. subtilis*, which inhibited Gram-positive pathogens and modulated host immune responses [5]. Furthermore, recent reports demonstrated that *Bacillus*-derived EPSs induced apoptosis and hindered cancer cell proliferation through diverse mechanisms [2,21].

This study aimed to valorize RBR and SBM for efficient EPS production using *B. tequilensis* PS21, employing RSM to optimize fermentation parameters, characterize EPS, and evaluate its bioactivity. Collectively, the findings from this study emphasize the feasibility and industrial relevance of producing multifunctional EPS from *B. tequilensis* PS21 using agro-waste substrates. This research highlights a cost-effective and eco-friendly strategy for developing multifunctional EPS suitable for high-value applications in food, cosmetics, and pharmaceuticals, while contributing to the circular bioeconomy.

## 2. Materials and Methods

### 2.1. RBR and SBM Substrates and Chemical Composition Analysis

The Agrarian Network of E-San Enterprise in Thailand’s Roi Et Province provided the RBR substrate. SBM was purchased at the Friends of Agriculture store in Kantarawichai District, Maha Sarakham Province, Thailand, as a source of nitrogen. After being ground into a fine 200 mm mesh, RBR and SBM were allowed to dry. The cellulose, hemicellulose, and lignin percentages were computed using the same methodology as previously reported [24]. The amounts of ash, moisture, and crude fat were measured using standard AOAC techniques as previously conducted [15]. The Kjeldhal approach was used to calculate the amount of crude protein.

### 2.2. Cultivation of EPS-Producing Bacteria

Initially, eight *Bacillus* bacteria from Thai milk kefir [19] were examined for their EPS synthesis utilizing RBR as a substrate. *Bacillus* spp. were cultivated in Tryptic Soy Broth (TSB) at pH 7.0 in an aerobic atmosphere for 24 h at 37 °C with agitation at 150 rpm. Prior to inoculation into flasks containing 5% (*w*/*v*) RBR with 100 mL of sterilized distilled water, the resulting suspension was adjusted to an optical density (OD_600_) of 0.1. This served as the medium for bacterial EPS synthesis. An inoculum of overnight bacterial culture at 3% (*w*/*v*) was introduced into the media and incubated aerobically at 37 °C with agitation at 150 rpm for 3 days. The reducing sugar content and pH values at 0, 1, 2, and 3 days were measured utilizing the DNS assay [25] and a pH meter. Bacterial growth was assessed by total plate count (TPC) on days 1 to 3, employing the spread plate method and serial dilution. The EPS content was ascertained on day 3, as previously documented [16].

### 2.3. Extraction of Crude EPS

Centrifugation was performed on the bacterial culture for 30 min at 16,100 g and 4 °C on the third day. Two liters of chilled 100% ethanol were used to retain the supernatant for 24 h at 4 °C without stirring. Two rounds of washing with 100% ethanol were performed on the EPS precipitate after centrifugation. The dry weight of the EPS was determined by first weighing it, and then drying it to a constant weight in an oven set at 40 °C. To remove the protein component, sterile distilled water was used to rehydrate 200 mg of dried crude EPS. Next, 20% trichloroacetic acid was added and left to sit at 4 °C for 1 h. After partially filtering the EPS with Vivaspin 10 kDa MWCO membrane ultrafiltration (Sartorius; Göttingen, Germany), the supernatant (EPS fraction) was freeze-dried. The weighed and dried crude EPS was then kept at −20 °C for bioassays. The EPS yield (g/L) was based on total fermentation broth volume.

### 2.4. Antioxidant Activity and Bioactive Compounds

The capacity of EPSs to absorb hydroxyl radicals was assessed [16]. To commence the reaction, 0.5 mL of EPS samples (20 mg/mL in deionized water), 0.5 mL of FeSO_4_ (0.8 mM), 0.5 mL of H_2_O_2_ (0.01% *v*/*v*), 1 mL of sodium phosphate buffer (0.2 M, pH 7.3), and 0.5 mL of 1,10-phenanthroline (0.8 mM) were combined in tubes and thoroughly mixed. The containers were incubated for 30 min at 37 °C. The absorbance of substances was measured at 536 nm.

Scavenging activity (%) = [1 − (A_sample_ − A_blank_)/A_control_] × 100

Absorbance of the blank (A_blank_) comprised deionized water, absorbance of the control (A_control_) included all reagents excluding the EPS sample, and absorbance of the sample (A_sample_) encompassed all reagents along with the EPS sample.

In addition, a previous report [23] detailed the DPPH scavenging assay. An EPS extract (20 μL) at a concentration of 20 mg/mL was mixed with 180 μL DPPH solution (10 mM) in methanol. After 30 min incubation in the dark, spectrophotometric measurements of the mixture’s absorbance were taken at 517 nm. The activity was calculated as above.

EPS (20 μL of a 20 mg/mL stock solution) was combined with a FRAP reagent (180 μL) containing 20 mM FeCl_3_, 10 mM 2,4,6-Tri (2-pyridyl) s-triazine, and a 0.3 M acetate buffer at pH 3.6, as previously reported [16]. The microplate reader recorded an absorption of 593 nm in triplicate, with measurements taken at 30 min intervals. Ferrous II sulfate was utilized as the standard.

The TPC was analyzed [16]. A total volume of 100 μL of 10% Folin–Ciocalteu solution was mixed with 20 μL of EPS (20 mg/mL) and 80 μL of 7.35% sodium carbonate. A measurement at 765 nm was recorded after a 30 min dark adaptation period. Gallic acid served as a reference standard.

TFC was formulated by mixing EPS (20 mg/mL) with deionized water (60 μL), 10% aluminum trichloride (10 μL), and 5% sodium nitrate (10 μL). After a 30 min reaction, the absorbance at 420 nm was measured following the addition of 100 μL of 1 M NaOH. A rutin standard was employed [16].

### 2.5. Antimicrobial Efficacy

An agar disk diffusion experiment was used to assess the antibacterial potential of EPS. *S. agalactiae* and *S. aureus*, the predominant pathogens in Nile tilapia production, were utilized to evaluate the antibacterial activity of EPSs. Eight *Bacillus* strains were cultivated overnight at 37 °C in Luria–Bertani (LB) broth, and the cultures were standardized to 10^8^ CFU/mL as measured by A_600nm_. A 0.22 μm membrane filter was used to sterilize EPS samples (20 mg/mL). One hundred microliters of bacterial suspensions were placed on LB agar plates. Paper disks (4 mm in diameter) were put on plates and 20 μL of EPS (20 mg/mL) was added to each disk. For 48 h, LB agar plates were incubated at 37 °C. The inhibitory zone diameter (mm) was used to measure the antibacterial efficacy.

### 2.6. Optimal Conditions for EPS Production Using RSM

Since EPS from *B. tequilensis* PS21 showed the most promising antioxidant and antimicrobial activities, it was chosen for the EPS production in RSM study. This study used the Central Composite Design (CCD) approach to develop and arrange trials in order to identify the ideal conditions for EPS synthesis. Four independent factors were studied: carbon source, nitrogen source, temperature, and pH. The first factor, the carbon source, was RBR powder (X1, 4–6%). The second factor, the nitrogen source, was SBM (X2 1–3%). The third factor was cultivation temperature (X3, 25–45 °C). The fourth factor was pH (X4, 4–7). The cultivation duration was fixed at 72 h at 150 rpm agitation speed. Experimental designs based on the Central Composite Design were created using the software Design–Expert Version 7, resulting in a total of 26 runs. These comprised five center points at levels −2, −1, 0, +1, and +2, four axial points, and four factorial (vertex) points (Table 1). Every point was examined three times. Polynomial regression analysis was utilized to build a mathematical model based on the data changes, and RSM using Design–Expert Version 7.00 (Stat-Ease, Inc., Minneapolis, MN, US) was used to assess the ideal circumstances for EPS production.

### 2.7. Purification of EPS

Crude EPS was extracted using the procedure previously mentioned above. A 5 mL HiTrap Q HP column (GE Healthcare, Hertfordshire, UK) was used for the purification process, which involved anion exchange chromatography with a starting buffer (25 mM Tris-HCl, pH 8.2) followed by elution with an elution buffer (25 mM Tris-HCl, 1.0 M NaCl, pH 8.2). The phenol–sulfuric acid method was used to measure the amount of carbohydrates (EPS) in the eluate (3.0 mL/tube). The EPS fractions eluted in a single peak were combined and dialyzed against ultrapure water, then concentrated via freeze-drying for subsequent analysis.

### 2.8. Characterization of EPS Structure

#### 2.8.1. Scanning Electron Microscopic (SEM) Analysis

The morphological properties of EPS were examined at 15 kV using an SEM (Leo/1450 Carl Zeiss, Oberkochen, Germany). An energy-dispersive X-ray analyzer (EDX) coupled to a scanning electron microscope (SEM) was used to investigate the elemental makeup of the EPS.

#### 2.8.2. Determination of EPS Molecular Weight

To ascertain the sample’s molecular weight distribution, gel permeation chromatography (GPC) was carried out utilizing a Nexera Series system (Shimadzu, Kyoto, Japan) fitted with a refractive index detector. Separation was achieved using a column set consisting of a TSKgel guardcolumn PWXL, TSKgel G5000PWXL (exclusion limit 2.5 × 10^6^ Da), TSKgel G4000PWXL (1 × 10^6^ Da), TSKgel G3000PWXL (2 × 10^5^ Da), and TSKgel G2500PWXL (5 × 10^3^ Da). The mobile phase used deionized water, operated under isocratic conditions at a flow rate of 0.6 mL/min. A 20 µL EPS sample volume was injected for each run. Pullulan standards with molecular weights ranging from 180 to 805,000 Da were used to calibrate the system. Prior to injection, 5 mg of the EPS sample was dissolved in 1 mL of deionized water and filtered through a 0.45 µm nylon 66 membrane filter to remove any particulates.

#### 2.8.3. Nuclear Magnetic Resonance (NMR) Analysis of EPS

Experiments were conducted on a JEOL JNM-ECZ-400R/S1 spectrometer functioning at a magnetic field strength of 9.39 T, translating to resonance frequencies of 100.5 MHz for ^13^C-NMR and 399.8 MHz for ^1^H-NMR. For the _13_C CP/MAS NMR experiment, approximately 57 mg of lyophilized EPS powder was packed into a 4 mm zirconia rotor with a Kel-F cap with a magic angle spinning (MAS) rate of 10 kHz. Cross-polarization from ^1^H to ^13^C was applied using 2 ms contact time, and proton decoupling was performed using the two-pulse phase modulation (TPPM) technique during acquisition. All 1440 scans were gathered, with a 5 s recycle delay, yielding high-resolution spectra. The ^13^C spectral window covered a sweep width of 63.13 kHz with an acquisition time of 32.44 ms. Data processing included exponential line broadening (60 Hz), zero-filling, Fourier transformation, phase correction, and fifth-order polynomial baseline correction. Spectral referencing was performed using the carbon peak of external adamantane at 38.5 ppm. For the ^1^H single pulse MAS NMR, the same sample was analyzed under identical spinning conditions (10 kHz MAS). Spectra were obtained with 720 scans, a sweep width of 250 kHz, and an acquisition time of 4.096 ms. A relaxation delay of 5 s was used between scans. The data were processed using exponential multiplication (20 Hz), Fourier transformation, and baseline correction. All spectral analyses were performed using JEOL Delta software v5.0.4.

#### 2.8.4. Fourier Transform Infrared (FTIR) Analysis

The functional groups of EPS were recorded using a Spectrum GX FTIR microscope (PerkinElmer Inc., Shelton, CT, USA). Following the 1:20 *w*/*w* pulverization of dehydrated EPS (1 mg) with KBr particles (20 mg), samples were FTIR scanned between 400 and 4000 cm^−1^.

#### 2.8.5. Monosaccharide Composition Via High-Performance Liquid Chromatography (HPLC) Analysis

Five milliliters of 2 M trifluoroacetic acid (TFA) were used to hydrolyze 100 mg of EPS in a sealed tube at 100 °C for 6 h. A 0.22 μm syringe filter was used to filter the hydrolysate after it had been neutralized with 1 N NaOH. The monomers of EPS were identified using HPLC (LC-20 AD, RID-10A refractive index detector, Shimadzu, Kyoto, Japan) equipped with an Aminex HPX-87H (Bio-Rad Laboratories, Hercules, CA, USA) carbohydrate analysis column at 65 °C, as was previously reported [13]. The mobile phase was H_2_SO_4_ (0.005 M) with an injection volume of 10 μL and a flow rate of 0.5 mL/min for 40 min per sample. Authentic monosaccharide sugar standards (including glucose, fructose, galactose, xylose, arabinose, and rhamnose), acquired from Sigma-Aldrich (St. Louis, MO, USA), were used to identify the sugar components in EPS using a UV detector to compare them with monosaccharide standards at 245 nm.

#### 2.8.6. X-Ray Diffraction (XRD) of EPS

Using a Co/Ka (λ = 1.7909 A0°) radiation source, the EPS powder was examined by XRD (Bruker, D8, Advance, Karlsruhe, Germany) in a 2Ɵ range from 5 to 80° at a scanning speed of 10°/min.

#### 2.8.7. Thermogravimetric Analysis (TGA) of EPS

Five milligrams of EPS was put in a platinum sample pan and analyzed using a NETZSCH TG 209 F3 TGA instrument (NETZSCH-Gerätebau GmbH, Selb, Germany). To avoid oxidative deterioration, the analysis was carried out in a nitrogen environment at a steady flow rate of 20 mL/min. At a linear heating rate of 10 °C/min, the sample was heated from 10 °C to 600 °C. The resulting thermograms were recorded as weight (%) versus temperature (°C). The onset of thermal degradation, temperature of maximum weight loss (T_max), and residual weight at 600 °C were determined using NETZSCH Proteus® software v9.0. Derivative thermogravimetry (DTG) is the first derivative of the TGA curve.

### 2.9. Determination of Bioactivities of EPS

#### 2.9.1. Cytotoxicity Assay

HeLa (cervical cancer cell line) and MCF-7 (breast cancer cell line) cells were grown in DMEM (high glucose) supplemented with 1% penicillin/streptomycin and 10% fetal bovine serum (FBS). The American Type Culture Collection in Manassas, Manassas, VA, USA, is where the cells were acquired. The cells were cultivated at 37 °C in an incubator with 5% CO_2_. Cells that reached around 80% confluence were extracted for cytotoxicity testing after 0.25% EDTA trypsin was added every three days when the medium was changed. In 96-well plates, 5000 HeLa or MCF-7 cancer cells were incubated for 24 h at 37 °C in 100 μL of DMEM containing 10% FBS. Cells were filled with diluted EPS extract in 100 μL at different concentrations for 24 h. Following the removal of the solutions, 100 μL of 3-[4,5-dimethylthiazol-2-yl]-2,5 diphenyl tetrazolium bromide (MTT) was added to the cells. After that, the mixture was let to sit for 4 h. After dissolving the formazan in 200 μL of dimethyl sulfoxide (DMSO), an absorbance at 590 nm was then measured in triplicate.

#### 2.9.2. Cell Morphology

A 24-well plate was planted with 7500 cancer cells/well in the medium. After overnight incubation, cells were added with different EPS doses for 24 h. Under an inverted microscope (NIB-100, Xenon, Ningbo, China), changes to cancer cell morphology were observed in triplicate.

#### 2.9.3. Clonogenic Assay

Following the methods outlined by Karirat et al. (2023) [15], this study examined colony development after treatment with plant extracts. Cancer cells were cultivated (500 cells/well) in DMEM for an entire night in a 6-well plate. Different dosages of EPS extract were applied to the cells for the duration of the treatment. The media was then removed, and a phosphate-buffered solution was used to rinse the cells. The incubation process was conducted at 37 °C for 14 days. Every two days, new media took its place. The cells were fixed in cold methanol for 30 min, and then they were stained for another 30 min with Coomassie brilliant blue g-250 (0.5% in methanol). After that, the colonies were counted. The trial was repeated three times.

#### 2.9.4. Wound Healing Assay

To grow cancer cells to 90% confluency, 2 × 10^5^ cells/well were introduced to the medium in a 24-well plate. A 200 µL pipette tip was then used to scrape the wound after cell debris had been cleaned with PBS. The cells were then exposed to EPS extracts (0, 25, 50, and 100 µg/mL) and allowed to incubate for the entire night. Cells were fixed with 4% formaldehyde and stained with 0.5% crystal violet for 30 min before being rinsed with distilled water. The percentage of scratch closure represents the reduction in the wound gap area over time, serving as an indicator of cell migration and proliferation during wound healing. The percentage of scratch closure was calculated as follows:

The percentage (%) of scratch closure = [(wound width at 0 h − wound width at 24 h)/(wound width at 0 h)] × 100

#### 2.9.5. Anti-Tyrosinase Activity

In short, 50 µL of different EPS sample concentrations and 100 µL of mushroom tyrosinase (100 U/mL in 20 mM phosphate buffer, pH 6.8) made up the reaction mixture (200 µL), which was put into the wells of a 96-well microplate. Following 15 min of pre-incubation at room temperature, 50 µL of 16 mM L-DOPA was added, and the mixture was then incubated for 30 min at 37 °C. A microplate reader (Spark Multimode, Tecan Group, Männedorf, Switzerland) was used to measure the amount of dopachrome that was produced at 492 nm. The tyrosinase inhibition (%) was calculated using the following equation:

Tyrosinase inhibition (%) = [(OD_control_ − OD_sample_)/OD_control_] × 100

where OD_control_ is the absorbance of the negative control (water in place of sample) and OD_sample_ is the absorbance of the sample.

#### 2.9.6. Anti-Collagenase Activity

Collagenase derived from *Clostridium histolyticum* was prepared in a 50 mM Tris-Cl buffer supplemented with NaCl and 5 mM CaCl_2_ to yield a working concentration of 0.0125 U/mL. DQ™ Gelatin (fluorescein-conjugated, sourced from pig skin; Thermo Fisher Scientific, Waltham, MA, USA) was also dissolved in the same buffer to a final concentration of 1 µg/mL. To conduct the assay, 40 µL of the EPS sample was mixed with 140 µL of enzyme buffer solution and pre-incubated for 15 min. Subsequently, 20 µL of the substrate solution was added to initiate the enzymatic reaction, bringing the total reaction volume to 200 µL. The mixture was then incubated at 37 °C for 30 min. Negative controls consisted of distilled water in place of the EPS. Fluorescence was recorded using a microplate reader (Spark Multimode, Tecan Group, Männedorf, Switzerland) at excitation and emission wavelengths of 485 nm and 535 nm, respectively. Collagenase inhibition (%) was calculated using the following equation:

Collagenase inhibition (%) = [(Fluorescence intensity_control_ − Fluorescence intensity_sample_)/Fluorescence intensity_control_] × 100

where fluorescence intensity_control_ is the fluorescence of the negative control (water in place of sample) and fluorescence intensity_sample_ is the fluorescence of the sample.

#### 2.9.7. Anti-Elastase Activity

The assay was conducted using 200 mM Tris-HCl buffer at pH 8.0. A stock solution of porcine pancreatic elastase (PE, E.C. 3.4.21.36) was prepared at 0.3 U/mL by dissolving the enzyme in sterile water. The substrate, N-Succinyl-Ala-Ala-Ala-p-nitroanilide (AAAPVN), was dissolved in the same buffer to a final concentration of 10 mM. To begin the assay, 50 µL of the EPS sample was combined with 100 µL of Tris-HCl buffer and 25 µL of the enzyme, followed by a 15 min incubation. The reaction was then initiated by adding 25 µL of the substrate solution, resulting in a total volume of 200 µL. The absorbance was read at 492 nm using a Spark Multimode microplate reader (Tecan Group, Männedorf, Switzerland). The elastase inhibition (%) was calculated using the following equation:

Elastase inhibition (%) = [(OD_control_ – OD_sample_)/OD_control_] × 100

where OD_control_ is the absorbance of the negative control (water in place of sample) and OD_sample_ is the absorbance of the sample.

### 2.10. Statistical Analysis

All results are presented as the mean ± standard deviation (SD). Statistical analysis was conducted using SPSS Version 20, employing one-way analysis of variance (ANOVA) based on a completely randomized design (CRD), with significance between means assessed using Duncan’s Multiple Range Test (DMRT) at a 95% confidence level (*p* < 0.05). To model the data, polynomial regression analysis was used, and the optimal parameters for EPS production were identified using RSM Via Design–Expert Version 7.00 software (Stat-Ease, Inc., Minneapolis, MN, USA).

## 3. Results and Discussion

### 3.1. Chemical Composition of RBR and SBM

The chemical compositions of RBR and SBM are presented in Table 2. RBR exhibited a moisture content of 11.73 ± 0.13%, ash content of 1.42 ± 0.01%, and fat content of 2.49 ± 0.07%. Its protein and fiber contents were relatively low, measured at 8.33 ± 0.15% and 0.96 ± 0.16%, respectively. Notably, RBR showed a high nitrogen-free extract (NFE) value of 75.06 ± 0.24%, reflecting its richness in fermentable carbohydrates such as starches and sugars, thereby supporting its potential as a carbon source in microbial fermentation.

In contrast, SBM contained 10.11 ± 0.05% moisture, with significantly higher ash (6.62 ± 0.01%) and protein (44.80 ± 0.02%) levels, confirming its suitability as a nitrogen-rich supplement for microbial growth. Its fiber content was higher than RBR at 6.07 ± 0.04%, while the NFE was comparatively lower at 31.17 ± 0.01%, suggesting a lower fermentable sugar content. SBM also exhibited higher acid detergent fiber (7.67 ± 0.19%) and moderate hemicellulose content (6.24 ± 0.13%). The complementary composition of RBR and SBM—high carbohydrates versus high protein, respectively—demonstrates their potential synergy as co-substrates in optimizing microbial metabolite production processes such as EPS biosynthesis. These characteristics are in line with previous studies where agro-residues, such as cassava pulp and sugarcane molasses, were successfully employed as low-cost substrates for EPS biosynthesis by *Bacillus* species [4,26]. Such valorization strategies reduce production costs and contribute to sustainable bioprocessing frameworks.

### 3.2. EPS Production from RBR Substrate by Bacillus spp.

**Figure 1:**
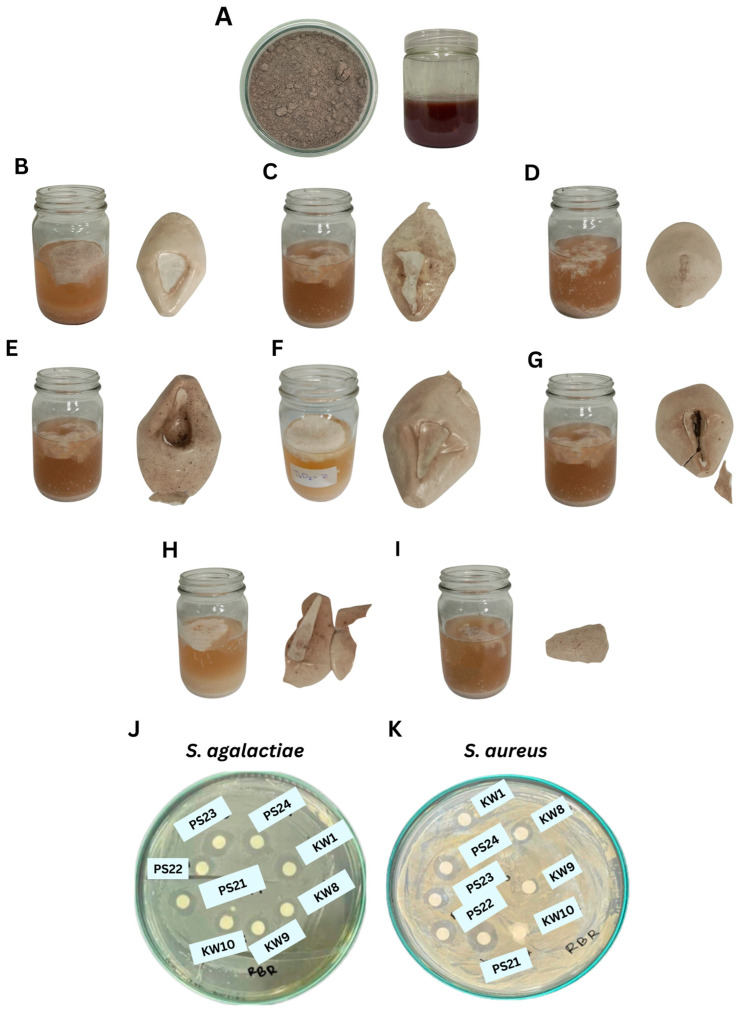
Crude EPSs from RBR substrate by *Bacillus* spp. and their antimicrobial activities. (**A**) RBR media on day 0, (**B**) *B. amyloliquefaciens* KW1, (**C**) *B. amyloliquefaciens* KW8, (**D**) *B. amyloliquefaciens* KW9, (**E**) *B. amyloliquefaciens* KW10, (**F**) *B. tequilensis* PS21, (**G**) *B. tequilensis* PS22, (**H**) *B. teqilensis* PS23, and (**I**) *B. tequilensis* PS24. (**J**) Antimicrobial activity of crude EPS extract against *S. agalactiae* and (**K**) *S. aureus*.

EPS production using eight *Bacillus* strains grown on RBR medium exhibited significant variation (*p* < 0.05) in yield over a three-day cultivation period (Table 3, Figure 1A–I). Among all strains, *B. tequilensis* PS21 demonstrated the highest EPS production, yielding 17.91 ± 1.70 g fresh weight (FW)/100 mL and 2.54 ± 0.14 g dry weight (DW)/100 mL. This was followed closely by *B. amyloliquefaciens* KW1 (2.17 ± 0.21 g DW/100 mL) and KW10 (1.97 ± 0.10 g DW/100 mL).

In parallel, PS21 maintained the highest cell viability as indicated by the total plate count (TPC) of 9.66 ± 0.02 log CFU/mL by day 3, exceeding that of other strains such as KW1 (9.63 ± 0.10 log CFU/mL). The pH values across all fermentations declined slightly over time—from ~6.7 on day 1 to ~6.4 on day 3—reflecting the accumulation of organic acids, a metabolic hallmark of *Bacillus* species during carbohydrate-rich fermentation [27]. The consumption of reducing sugars followed a typical trend, with levels increasing from day 1 to day 2 and declining by day 3, corresponding with active sugar metabolism and EPS biosynthesis. Strains with a higher sugar uptake, such as PS21 and KW1, also exhibited elevated EPS production, reinforcing the correlation between substrate utilization and polysaccharide output.

EPS production among the tested strains in this study compared favorably with previous reports that utilized commercial substrates. For instance, *B. amyloliquefaciens* RT7 produced ~2.8 g/L EPS in glucose–Tween medium [27], while *B. xiamenensis* RT6 achieved 2.3–2.7 g/L in acidic waste-based media [28]. Similarly, *B. licheniformis* MS3 yielded up to 3.4 g/L EPS from wheat bran extract under optimized conditions [4] and *B. albus* DM-15 produced EPS from Dasamoolarishta fermentation with demonstrated antioxidant and anticancer activities [22]. However, the present study uniquely achieves a higher EPS yield using agro-industrial residues (RBR and SBM) without supplementation with refined sugars or commercial media components, highlighting the cost effectiveness and sustainability of this bioprocess. Overall, these findings underscore *B. tequilensis* PS21 as a promising EPS-producing candidate under agro-waste-based fermentation, with potential for scale-up in industrial biopolymer production systems.

### 3.3. Antioxidant Properties and Levels of Bioactive Compounds

In terms of antioxidant properties, the EPS extracted from *B. tequilensis* PS21 showed superior activity across all assays (*p* < 0.05) (Table 4).

*B. tequilensis* PS21 EPS exhibited the highest DPPH radical scavenging activity (65.50 ± 0.31%), hydroxyl radical scavenging activity (80.53 ± 0.87%), and ferric reducing antioxidant power (FRAP: 6.51 ± 0.10 mg Fe^2+^/g DW). The EPS extract exhibited a significantly higher total phenolic content (TPC: 17.98 ± 0.57 mg GAE/g DW) and total flavonoid content (TFC: 39.14 ± 0.33 mg RE/g DW). These bioactive compounds may serve as redox-active moieties within the EPS structure, potentially contributing to its markedly superior antioxidant capacity compared with the EPSs produced by other strains. These findings align with earlier studies on *Bacillus*-derived EPSs. For instance, *B. subtilis* AG4 was reported to produce EPSs with strong DPPH and ABTS scavenging activity linked to anti-aging and cytoprotective effects [1]. Similarly, EPS from *B. albus* DM-15 demonstrated comparable ROS scavenging properties [22], reinforcing the relevance of such polymers in health-promoting applications. The results in this work surpass those reported for *B. tequilensis* FR9 EPS, which demonstrated a hydroxyl radical scavenging activity of 76.95% at 4 mg/mL [29] and for *B. amyloliquefaciens* C-1 EPS showing a DPPH radical scavenging activity of 60.4% [30]. The strong antioxidant capacity of PS21 EPS highlights its potential applications not only in nutraceuticals, but also in cosmetics, where there is a growing demand for agents that protect against oxidative stress and delay skin aging.

In terms of antimicrobial activity, EPSs showed varying degrees of inhibition against *S. agalactiae* and *S. aureus* (Table 4, Figure 1J). The EPS from *B. tequilensis* PS23 demonstrated the strongest inhibition against *S. agalactiae* (11.28 ± 0.54 mm), whereas PS21 showed broad-spectrum activity with clear zones of 10.07 ± 0.61 mm and 7.83 ± 0.76 mm against *S. agalactiae* and *S. aureus*, respectively.

The antimicrobial activity of EPS (20 mg/mL) produced using *B. tequilensis* PS21 and other *Bacillus* strains against *S. aureus* was evaluated and compared with EPSs from lactic acid bacteria (LAB) reported in previous studies. In this study, PS21 EPS exhibited a strong inhibitory effect against *S. aureus*, with an inhibition zone of 7.83 ± 0.76 mm. This performance is comparable to or exceeds several LAB-derived EPSs reviewed by Abdalla et al. (2021) [31], where inhibition zones for *S. aureus* typically ranged from 2 mm to 10.2 mm depending on the strain and EPS characteristics. For instance, EPS produced by *Lactobacillus gasseri* FR4 showed inhibition zones of 3.16 mm and 5.86 mm at 10 mg/mL against *S. aureus* [32]. The variation in antimicrobial efficacy among EPSs may be attributed to differences in molecular composition, charge density, and the presence of functional groups such as uronic acids and sulfates [33]. As Abdalla et al. (2021) [31] suggested, these structural features significantly influence the interaction of EPSs with bacterial cell walls, particularly the thick peptidoglycan layer of Gram-positive bacteria like *S. aureus*.

### 3.4. Optimized EPS Production from RBR and SBM Using B. Tequilensis PS21 Via CCD and RSM

Given the superior EPS yield and antioxidant properties demonstrated by *B. tequilensis* PS21, this strain was selected for optimization studies using CCD and RSM. RBR and SBM served as low-cost carbon and nitrogen sources, respectively, while pH and temperature were additional independent variables. A total of 26 experimental runs were conducted to evaluate the interactive effects of these variables on EPS production and bacterial growth (Table 5).

From Table 5, the investigation into the factors influencing EPS production revealed that variations in carbon source, nitrogen source, temperature, and pH significantly affected EPS yield (*p* < 0.05). As the fermentation time progressed, EPS production increased in parallel with bacterial growth (Table 5). A polynomial regression analysis was applied to the experimental data to develop a predictive model for EPS production and to identify optimal conditions through RSM. The resulting equation representing the EPS production model is

EPS (g/L) = +37.82 − 1.98A + 3.35B + 1.16C + 3.45D − 1.02AB − 0.61AC − 1.39AD + 0.75BC + 2.23BD + 0.25CD − 2.16A^2^ − 1.99B^2^ − 5.26C^2^ − 3.18D^2^

where A = RBR (%), B = SBM (%), C = pH, and D = temperature (°C).

From the regression equation, it can be observed that all four independent variables (A, B, C, and D) exert positive linear effects on EPS production, as indicated by their positive coefficients. This implies that increasing any of these factors within the studied range contributes to an increase in EPS yield.

Among the runs, the highest EPS yield (38.70 g/L) and bacterial count (12.86 log CFU/mL) were obtained under unmodified central point conditions (runs 7 and 25), indicating the suitability of the selected variable ranges. Conversely, the lowest EPS yield (13.40 g/L) occurred at a strongly acidic pH (run 16), confirming that suboptimal pH levels can drastically inhibit production.

**Figure 2:**
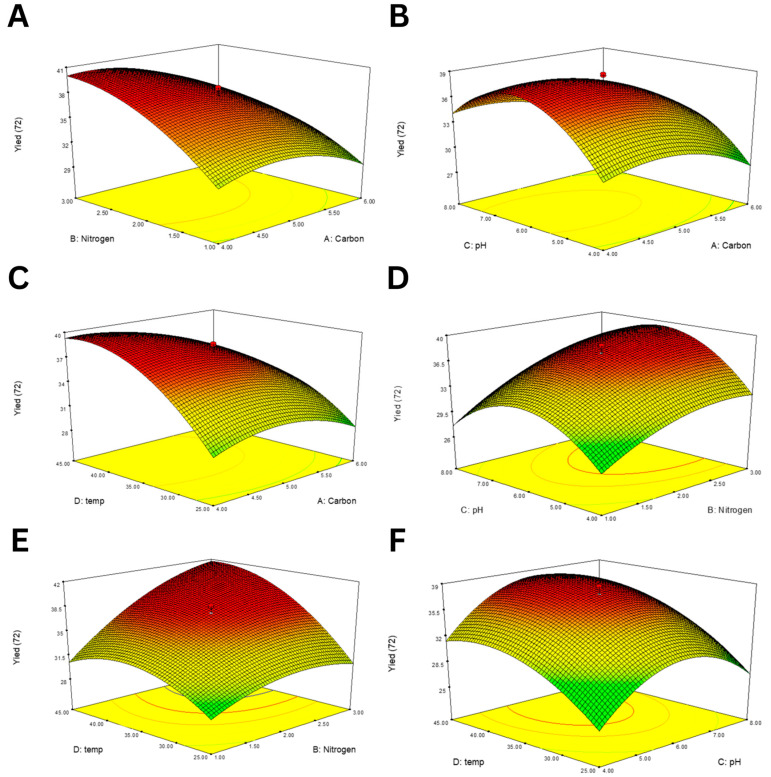
RSM plots showing the interaction effects of various factor pairs on EPS production using *B. tequilensis* PS21. (**A**) RBR and SBM, (**B**) RBR and pH, (**C**) RBR and temperature, (**D**) SBM and pH, (**E**) SBM and temperature, and (**F**) temperature and pH.

The 3D response surface plots (Figure 2) illustrate the interaction effects of each pair of variables on EPS yield. A positive interaction was observed between RBR and SBM concentrations (Figure 2A), suggesting that a balanced supply of both carbon and nitrogen enhances production. Similar synergistic patterns were observed between RBR and pH (Figure 2B) and SBM and temperature (Figure 2E). In contrast, extreme values of pH or temperature negatively influenced yield, indicating narrow optimal ranges for both parameters (Figure 2F,C).

The ANOVA results (Table 6) confirmed that the quadratic model for EPS production was highly significant (*p* < 0.0001) with an F value of 37.21. Individual factors, including nitrogen (B), temperature (D), and the quadratic effects of all four variables (A^2^, B^2^, C^2^, and D^2^), had strong effects (*p* < 0.01). No significant lack of fit was observed (*p* = 0.0518), and the model exhibited a strong predictive performance, as indicated by R^2^ = 0.9793, adjusted R^2^ = 0.9530, and an adequate precision of 18.12.

**Figure 3:**
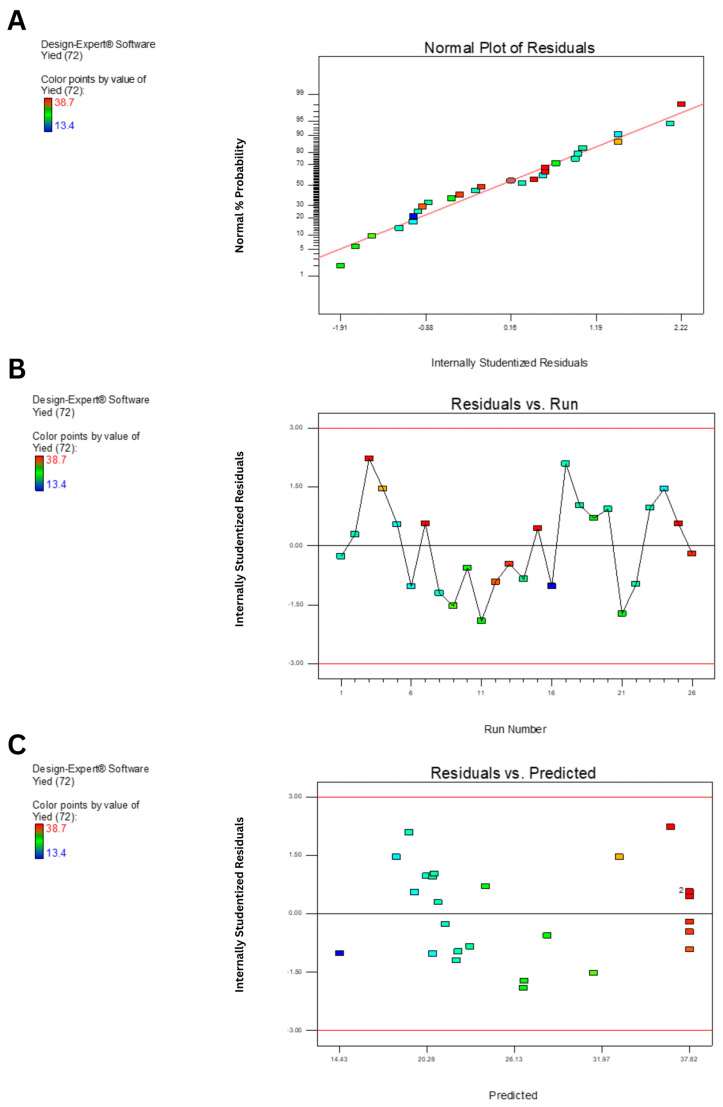
Diagnostic plots for model validation of EPS yield prediction using *B. tequilensis* PS21 using RBR and SBM as substrates. (**A**) Normal plot of residuals. (**B**) Plot of internally studentized residuals versus run number. (**C**) Plot of internally studentized residuals versus predicted values.

Diagnostic plots (Figure 3) further validated the model adequacy. The normal plot of residuals (Figure 3A) showed points closely aligned to the straight line, confirming the normality of residuals. The residuals vs. run plot (Figure 3B) exhibited random scatter, indicating independence. Meanwhile, the residuals vs. predicted values plot (Figure 3C) showed homoscedasticity, supporting the assumption of constant variance.

The numerical optimization process identified multiple optimal conditions for maximum EPS production with high desirability scores (0.956–0.977) (Table 7). The highest predicted EPS yield was 41.17 g/L at 5% (*w*/*v*) RBR, 3% (*w*/*v*) SBM, pH 6.66, and 39.51 °C. Experimental validation under these conditions resulted in an actual EPS yield of 39.82 ± 0.03 g/L, with a mean difference of only 1.36 g/L from the predicted value. This EPS yield represented a 15-fold increase compared with the yield obtained from RBR alone before the application of RSM.

Other validated runs also yielded consistent results with minimal deviation, confirming the robustness of the model.

Overall, RSM optimization effectively enhanced EPS production using *B. tequilensis* PS21 using cost-effective agro-industrial substrates. The optimized yield (≈39–40 g/L) surpassed previously reported yields from similar *Bacillus* species using synthetic or refined media, underscoring the potential of this system for sustainable and economical biopolymer production.

### 3.5. Structural and Physicochemical Characterization of the Purified EPS

**Figure 4:**
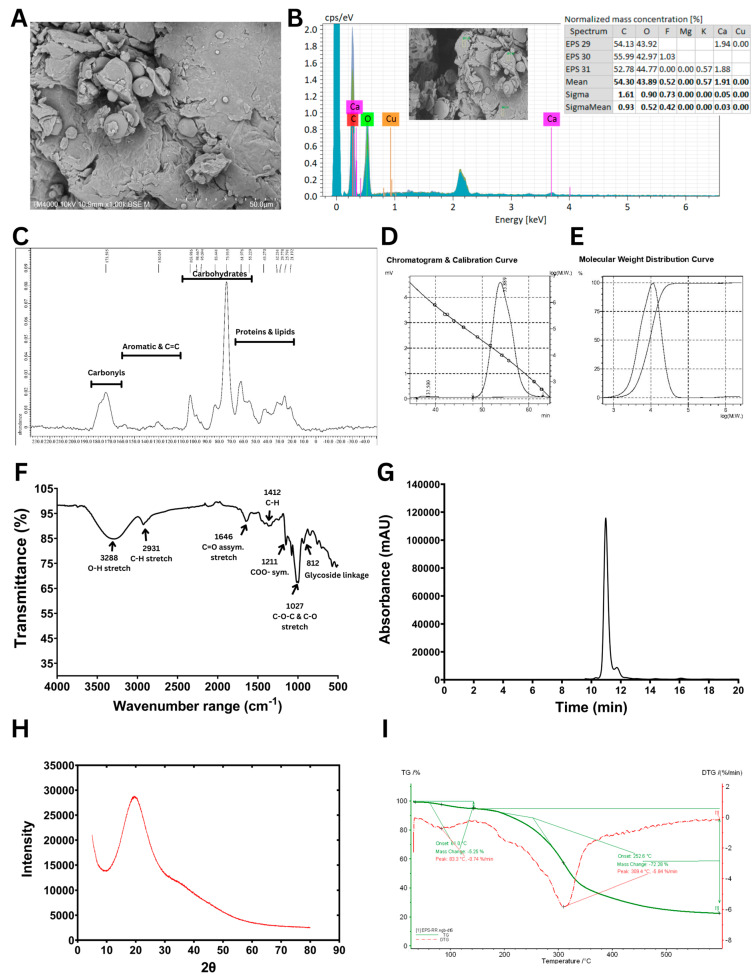
Structural and physicochemical characterization of EPS synthesized using *B. tequilensis* PS21 cultivated under optimal conditions (5% (*w/v*) RBR, 3% (*w/v*) SBM, pH 6.66, and 39.51 °C) for 3 days. (**A**) SEM image. (**B**) SEM-EDX data. (**C**) Solid-state ^13^C NMR spectrum. (**D**) GPC chromatogram and calibration curve. (**E**) Molecular weight distribution curve. (**F**) FTIR spectrum. (**G**) HPLC profile. (**H**) XRD pattern. (**I**) TGA and DTG curves.

The structural and physicochemical characteristics of the purified *B. tequilensis* PS21 EPS under optimized conditions based on RBR and SBM substrates were comprehensively analyzed using multiple analytical techniques (Figure 4).

The SEM analysis (Figure 4A) revealed that *B. tequilensis* PS21 EPS exhibits an irregular, uneven surface with loosely packed, layered flakes and interstitial spaces, suggesting a non-crystalline, porous polymeric matrix. Such porous EPS structures enhance water retention via hydrogen bonding, making EPSs suitable for use as thickeners, texturizers, and stabilizers in food systems [34]. Similar web-like architectures with stacked flake-like polysaccharides have been reported in EPSs from *B. licheniformis* PASS26, contributing to improved porosity and functional properties [35]. Beyond food applications, porous EPSs like those from *B. atrophaeus* WYZ and *Rhodosporidium babjevae* have shown promise in drug delivery and as biocompatible excipients in pharmaceuticals [36,37]. These findings highlight the versatility of porous EPSs for diverse industrial uses.

The EDX data (Figure 4B) revealed that carbon (C) and oxygen (O) were the predominant elements, with mean normalized mass concentrations of 54.30% and 43.89%, respectively, indicating the organic and polysaccharidic nature of the sample. Trace elements were negligible. The absence of heavy metals and the presence of only biocompatible elements further support the suitability of this EPS for food, pharmaceutical, and biomedical applications. The elemental composition observed is consistent with previous studies on microbial EPSs, which commonly exhibit a high carbon and oxygen content due to their polysaccharide backbone, and a variable inorganic content depending on the microbial source and culture conditions [38].

The solid-state ^13^C CP/MAS NMR spectrum of the EPS (Figure 4C) revealed key resonances consistent with a sugar-based polymer. Similar to the previous report of the solid-state ^13^C CP/MAS NMR spectrum of biofilm from *B. subtilis* [39], a prominent signal at 98.5 ppm was observed, characteristic of the anomeric carbon (C1) in hexose units, indicating glycosidic linkage. A series of peaks in the 70–75 ppm range were attributed to carbons C2–C5 in glucose or mannose units bonded to oxygen (C–O). Additionally, a signal at 173.4 ppm was noted, corresponding to carbonyl groups (C = O), which are indicative of carboxyl, ester, or amide functionalities, possibly due to natural modifications or the derivatization of the polysaccharide backbone. A minor cluster of peaks in the 20–30 ppm region pointed to the presence of aliphatic carbons, suggesting possible non-carbohydrate substituents.

The GPC analysis of the EPS (Figure 4D,E) revealed a single, symmetrical elution peak at 53.9 min, corresponding to a homogeneous polymer. The weight-averaged molecular weight (M_w_) was 11.3 × 10^3^ Da, while the number-averaged molecular weight (M_n_) was 7.6 × 10^3^ Da, yielding a polydispersity index (M_w_/M_n_) of 1.47. This reflects a moderately uniform molecular size distribution. Notably, the molecular weight of this EPS is relatively low compared with many previously reported microbial EPSs. For example, Wang et al. (2020) [40] showed that levan from *Bacillus* exhibited a M_w_ of 1.56 × 10^6^ Da. Similarly, Raga-Carbajal et al. (2018) [41] identified both high- and low-molecular-weight (2.3 × 10^6^ Da and 7.2 × 10^3^ Da, respectively) levans produced using *B. subtilis*. Xu et al. (2016) [42] further highlighted the variability of levan molecular weights across strains and conditions, noting that *B. subtilis* produced levans ranging from 11 kDa to 1800 kDa, whereas *B. polymyxa* (NRRL B-18475) synthesized levans as low as ~2.1 × 10^3^ Da. By comparison, Farag et al. (2020) [43] found that *Bacillus mycoides* BS4 synthesized a heteropolysaccharide predominantly consisting of glucose, accompanied by galactose, mannose, and glucuronic acid, with 1.90 × 10^4^ Da molecular weight. Although the EPS characterized in the present study (11.3 kDa) has a lower molecular weight than that of *B. mycoides* BS4, both fall within the low-molecular-weight category. Such EPSs are often associated with enhanced biological properties, including antitumor and immunomodulatory activities. These differences in the molecular weight of EPSs are commonly attributed to microbial strain specificity, fermentation conditions, carbon source availability, and extraction techniques [14].

The FTIR spectrum of the EPS (Figure 4F) indicated the distinctive functional groups of carbohydrates. The IR spectral profile was consistent with those reported for known polysaccharides, allowing the interpretation of characteristic peaks based on the prior literature [38]. A broad absorption band centered at 3288 cm^−1^ was observed, corresponding to the O–H stretching vibrations typically found in polysaccharide structures. The band at 2931 cm^−1^ was attributed to the C–H stretching of aliphatic groups, further confirming the carbohydrate nature of the sample. An absorption peak at 1646 cm^−1^ indicated asymmetric C = O stretching, which may be associated with uronic acid components or hydrogen-bonded carbonyl groups. Signals in the 1400–1200 cm^−1^ range, particularly the band at 1412 cm^−1^, were linked to C–H bending and symmetric COO^−^ stretching, often present in carboxylated polysaccharides. The strong absorption region between 1150 and 975 cm^−1^, featuring a distinct peak at 1027 cm^−1^, corresponded to the C–O and C–O–C stretching vibrations of glycosidic bonds, confirming the presence of polysaccharide linkages in the EPS. Additionally, the peak at 812 cm^−1^ corresponds to the presence of α-glycosidic linkages, further supporting the carbohydrate configuration of the biopolymer. These spectral features align with previously reported FTIR patterns of bacterial EPS such as those from *Paenibacillus bovis* and *B. megaterium* [42,44].

The HPLC chromatogram (Figure 4G) confirmed that glucose was the sole monosaccharide detected following acid hydrolysis, indicating that the EPS is a homopolysaccharide. This finding is consistent with both the NMR and FTIR analyses, which collectively support the identification of the EPS as a glucose-based polysaccharide featuring α-anomeric glucosidic linkages.

The X-ray diffraction (XRD) pattern of the EPS (Figure 4H) displayed a broad and diffuse peak centered at approximately 2θ ≈ 20°, a hallmark of amorphous polysaccharides [45], supporting the amorphous structure observed in the SEM image (Figure 4A). Likewise, the EPS from *Bacillus* sp. EPS003 also showed an amorphous structure [46].

Finally, the TGA and DTG curves (Figure 4I) revealed that the EPS maintained thermal stability up to approximately 250 °C, with the primary degradation phase occurring between 250 and 350 °C. The high onset degradation temperature and the presence of around 25% residual mass at 600 °C indicate good thermal resistance, suggesting its suitability for high-temperature industrial applications. These thermal properties are consistent with EPSs derived from *Enterobacter* sp. and *Bacillus* sp., which showed potential usage as fillers or components in composite films for applications such as food packaging and biomedical materials [47].

In summary, the EPS from *Bacillus tequilensis* PS21 is a structurally homogeneous, thermally stable, and biofunctionally favorable polymer composed primarily of glucose. It is characterized by an amorphous morphology and well-defined glycosidic linkages, attributes that underscore its potential for applications in the food, cosmetic, and biomedical industries. These structural and physicochemical properties are consistent with previous studies on Bacillus-derived EPSs. For instance, Vinothkanna et al. (2022) [22] reported that the EPS from the probiotic *Bacillus albus* DM-15 exhibits a non-crystalline architecture, thermal stability, and multifunctional bioactivities, including antioxidant, emulsifying, and cytotoxic effects against cancer cell lines. Similarly, Sathishkumar et al. (2021) [20] comprehensively characterized an EPS from sponge-associated *Bacillus subtilis* MKU SERB2 using SEM, XRD, FTIR, ^1^H/^13^C NMR, and EDX. This EPS displayed a fibrous, porous, semi-crystalline morphology and contained primarily carbon and oxygen along with trace elements. It also demonstrated notable antioxidant and anticoagulant activities without exhibiting cytotoxicity, suggesting its suitability for food and pharmaceutical applications. Collectively, these findings reinforce the potential of non-crystalline, glucose-rich EPSs such as that from *B. tequilensis* PS21 as promising biopolymers for diverse industrial uses.

### 3.6. Bioactivities of EPS

**Figure 5:**
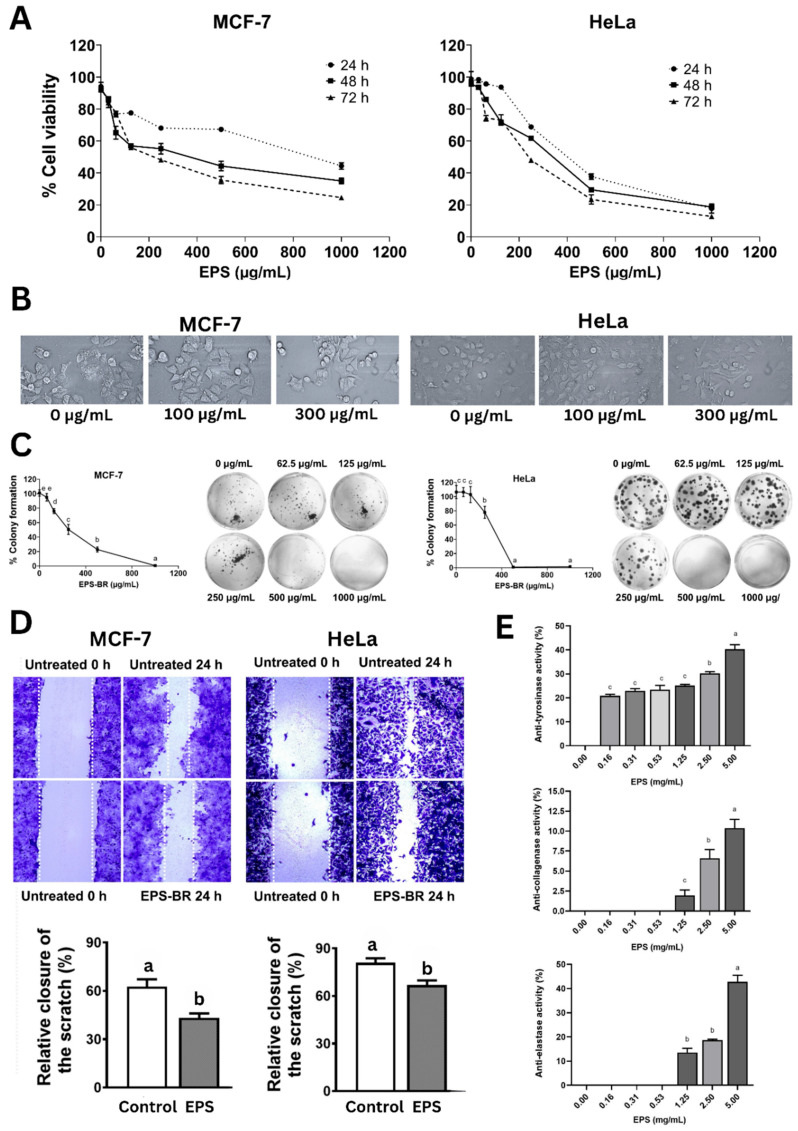
Bioactivities of EPS. (**A**) Cytotoxicity of EPS on MCF-7 and HeLa over time. (**B**) Cell morphology of MCF-7 and HeLa cells after treatment with EPS (100 µg/mL) over 24 h. (**C**) Colony formation of MCF-7 and HeLa cells upon EPS treatment. (**D**) Wound healing assay of MCF-7 and HeLa cells upon EPS treatment over 24 h. (**E**) Anti-tyrosinase, anti-collagenase, and anti-elastase activities of EPS doses. Statistical significance at *p* < 0.05 is indicated by different lowercase letters.

The cytotoxicity of the EPS extract on MCF-7 (breast cancer) and HeLa (cervical cancer) cell viability were assessed at 24, 48, and 72 h (Table 8, Figure 5A).

The cytotoxicity assay (Figure 5A) showed a dose- and time-dependent decline in cell viability, with IC_50_ values decreasing over time, aligning with previous findings on EPS from *B. subtilis* and *B. velezensis* that induced apoptosis via mitochondrial and cell cycle arrest mechanisms [2,48]. With MCF-7, cytotoxicity increased from 55.61% to 75.44% and IC_50_ values dropped from 1069.23 to 226.60 µg/mL over 72 h. Similarly, HeLa cells showed increased cytotoxicity from 82.26% to 87.24%, with IC_50_ decreasing from 396.83 to 224.30 µg/mL, indicating more progressive anticancer effects, especially at 72 h, and that the EPS was more effective against HeLa than MCF-7.

Compared with the findings of Selim et al. (2022) [49], where the EPS (glucose, galacturonic acid, and arabinose monomers) derived from marine *B. cereus* exhibited strong cytotoxicity against MCF-7 human breast carcinoma cells (IC_50_ = 55.7 ± 2.3 μg/mL), our EPS showed a relatively lower activity against the same cell line. However, our EPS demonstrated greater cytotoxic potency against HeLa cells when compared with the EPS consisting of D-mannose, D-glucose, and L-fucose from *Enterococcus faecalis* reported by Choudhuri et al. (2020) [50], which had an IC_50_ value of 267.3 µg/mL.

The morphological changes in both cancer cells with increasing EPS doses such as cell shrinkage and detachment and also a lower cell density (Figure 5B) further support apoptotic processes [51]. EPS treatment also significantly inhibited colony formation (Figure 5C), a key indicator of long-term survival. EPS inhibited colony formation in both MCF-7 and HeLa cells in a concentration-dependent fashion. Notably, colony formation dropped below 40% at 250 µg/mL in MCF-7 cells and was completely suppressed at 1000 µg/mL, while HeLa cells showed complete inhibition from 500 µg/mL onward, indicating strong antiproliferative effects (Figure 5C). Additionally, EPS significantly impaired cell migration in wound healing assays (Figure 5D), which parallels anti-metastatic effects. EPS substantially minimized cancer cell migration in both MCF-7 and HeLa cells, lowering wound closure by approximately 20–25% compared with untreated controls after 24 h, indicating a strong anti-migratory effect. Our results are in agreement with those of Xiang et al. (2021) [52], who found that exopolysaccharides produced by *Rhizopus nigricans* inhibited the proliferation of MCF-7 breast cancer cells in a dose-dependent fashion. Their study reported notable decreases in cell viability at 48 and 72 h, with IC_50_ values of 387.1 µg/mL and 357.2 µg/mL, respectively. Moreover, EPS treatment markedly suppressed cell migration, as evidenced by reduced wound closure in scratch assays over the same time periods, further confirming its potential to inhibit MCF-7 cell motility in a concentration-dependent manner.

Throughout history, humans have pursued ways to prolong life and preserve youthful appearances, frequently relying on natural compounds to achieve these goals. In this context, the present study evaluated an exopolysaccharide (EPS) for its inhibitory effects on key skin-aging enzymes—tyrosinase, collagenase, and elastase. The EPS demonstrated significant dose-dependent inhibition across all three enzymes (Figure 5E). Specifically, anti-tyrosinase activity increased from 25.63% at 0.16 mg/mL to 38.88% at 5.00 mg/mL after 30 min of incubation, with statistically significant differences observed at concentrations ≥2.5 mg/mL. However, its tyrosinase inhibition was lower compared with the EPS derived from *Streptomyces* sp. NRCG4, which showed 25.39% inhibition at 0.4 mg/mL in 10 min and reached 75.12% after 20 min of incubation [53].

Likewise, collagenase inhibition rose modestly from 2.43% to 12.96% as the EPS doses rose from 0.16 to 5.00 mg/mL (Figure 5E). In contrast, elastase inhibition demonstrated a more pronounced dose-dependent effect, climbing from 6.87% at 1.25 mg/mL to 42.36% at 5.00 mg/mL, representing medium activity (Figure 5E). These findings align with those reported by Shirzad et al. (2018) [54], who showed that EPS from several *Lactobacillus* strains, such as *L. plantarum* P8-1 and C6-3, *L. helveticus* Y8-1, *L. fermentum* B6-1 and B2-2, and *L. rhamnosus* YC-1, exhibited elastase inhibition ranging from 20% to 50%. On the other hand, *L. delbrueckii* Y7-1 and *L. curvatus* C8-1 showed minimal inhibition (~5%). Similarly, low anti-collagenase activity (2–20%) was reported in strains such as *L. plantarum* P35, *L. delbrueckii* Y7-1, and *L. fermentum* C1-3, aligning with the limited collagenase inhibition observed in our study.

Further evidence for the dermatological potential of EPS is provided by Touni et al. (2024) [7], who reported that EPSs derived from *B. subtilis* significantly suppressed depigmentation in a vitiligo model, suggesting their applicability in skin whitening and pigmentation regulation. From an industrial standpoint, the EPS’s thermal stability, amorphous morphology, and antioxidant capacity enhance its applicability in pharmaceuticals, nutraceuticals, and personal care products. Collectively, these results underscore the broad-spectrum bioactivities of the EPS, establishing it as a promising multifunctional biopolymer for anticancer and skin care formulations.

## 4. Conclusions

This study demonstrates the successful valorization of agro-industrial by-products—RBR and SBM—as cost-effective substrates for enhanced EPS production using *B. tequilensis* PS21. The EPS produced from RBR alone showed notable bioactivities, including strong antioxidant properties (65.5% DPPH scavenging, 80.5% hydroxyl radical scavenging, and 6.51 mg Fe^2+^/g FRAP) and antimicrobial effects against *S. agalactiae* and *S. aureus*. Optimization through RSM, employing RBR and SBM as co-substrates, increased EPS yield to 39.82 g/L—a 15-fold improvement over unoptimized conditions. The optimized EPS was characterized as a glucose-rich, structurally homogeneous, and thermally stable polymer with an amorphous morphology. Functionally, it exhibited anticancer activity against MCF-7 and HeLa cells (IC_50_ ≈ 224–227 µg/mL at 72 h) along with antiproliferative and anti-migratory effects. Additionally, its inhibition of tyrosinase, collagenase, and elastase underscores its potential as a bioactive ingredient in cosmetic formulations targeting skin aging and hyperpigmentation. These findings highlight the cost-effective and eco-friendly innovation of coupling agro-waste valorization with bioprocess optimization to produce multifunctional EPSs with broad applications in food preservation, pharmaceutical excipients, nutraceuticals, and cosmeceuticals. This sustainable approach aligns with the BCG economic model, demonstrating how local bioresources can be transformed into high-value biopolymers that support both environmental and industrial goals.

## Figures and Tables

**Table 1:** Independent variables of central composite design (CCD) to produce EPS.

Independent Variables	Units	Levels	Variation Range
RBR (X_1_)	%	4–6	(−2, −1, 0, +1, and +2)
SBM (X_2_)	%	1–3	(−2, −1, 0, +1, and +2)
Temperature (X_3_)	°C	25–45	(−2, −1, 0, +1, and +2)
pH (X_4_)	-	4–7	(−2, −1, 0, +1, and +2)

**Table 2:** Chemical composition of substrates.

Substrate	Moisture%	Ash%	Fat%	Protein%	Fiber%	Starch and Sugar (%) (NFE)	%Hemi Cellulose (NDF-ADF)	% Cellulose + lignin (ADF)
Riceberry broken rice (RBR)	11.73 ± 0.13	1.42 ± 0.01	2.49 ± 0.07	8.33 ± 0.15	0.96 ± 0.16	75.06 ± 0.24	14.01 ± 0.41	1.43 ± 0.06
Soybean Meal (SBM)	10.11 ± 0.05	6.62 ± 0.01	1.26 ± 0.06	44.80 ± 0.02	6.07 ± 0.04	31.17 ± 0.01	6.24 ± 0.13	7.67 ± 0.19

%Nitrogen-Free Extract (NFE) = 100 − [%Moisture + %Ash+ %Protein + %Fat + %Fiber], Neutral detergent fiber (NDF), Acid detergent fiber (ADF).

**Table 3:** EPS synthesis from RBR substrate using *Bacillus* spp., pH, reducing sugar, and bacterial counts over 3 days.

Bacteria	EPS (g FW/100 mL)	EPS (g DW/100 mL)	pH Day 1 Day 2 Day 3	Reducing Sugar (mg/mL) Day 1 Day 2 Day 3	TPC (log CFU/mL) Day 1 Day 2 Day 3
*B. amyloliquefaciens* KW1	17.32 ± 2.03 ^a^	2.17 ± 0.21 ^a^	6.74 ± 0.02	0.967 ± 0.063	8.99 ± 0.02
			6.65 ± 0.02	2.175 ± 0.063	9.33 ± 0.05
			6.49 ± 0.05	1.711 ± 0.035	9.63 ± 0.10
*B. amyloliquefaciens* KW8	14.98 ± 2.91 ^b^	1.85 ± 0.08 ^b^	6.73 ± 0.06	0.800 ± 0.001	8.69 ± 0.02
			6.65 ± 0.04	1.465 ± 0.038	8.91 ± 0.01
			6.53 ± 0.07	1.191 ± 0.036	8.97 ± 0.01
*B. amyloliquefaciens* KW9	11.09 ± 1.09 ^c^	1.62 ± 0.20 ^c^	6.83 ± 0.03	0.742 ± 0.012	8.69 ± 0.01
			6.71 ± 0.07	1.102 ± 0.026	8.91 ± 0.01
			6.54 ± 0.03	0.919 ± 0.010	8.98 ± 0.01
*B. amyloliquefaciens* KW10	12.88 ± 0.44 ^c^	1.97 ± 0.10 ^b^	6.76 ± 0.09	0.059 ± 0.002	8.68 ± 0.10
			6.58 ± 0.09	1.466 ± 0.037	9.00 ± 0.01
			6.44 ± 0.08	1.187 ± 0.015	9.12 ± 0.00
*B. tequilensis* PS21	17.91 ± 1.70 ^a^	2.54 ± 0.14 ^a^	6.77 ± 0.07	0.970 ± 0.059	9.01 ± 0.01
			6.59 ± 0.02	2.141 ± 0.016	9.38 ± 0.03
			6.40 ± 0.09	1.702 ± 0.033	9.66 ± 0.02
*B. tequilensis* PS22	10.43 ± 0.11 ^c^	1.96 ± 0.07 ^b^	6.70 ± 0.06	0.735 ± 0.032	8.81 ± 0.01
			6.59 ± 0.07	1.468 ± 0.037	9.06 ± 0.01
			6.42 ± 0.05	1.140 ± 0.028	9.16 ± 0.00
*B. tequilensis* PS23	11.37 ± 0.74 ^c^	1.56 ± 0.09 ^c^	6.74 ± 0.04	0.612 ± 0.04	8.70 ± 0.01
			6.54 ± 0.10	1.077 ± 0.031	8.93 ± 0.03
			6.35 ± 0.09	0.803 ± 0.022	8.99 ± 0.00
*B. methylotrophicus* PS24	10.50 ± 0.14 ^c^	1.69 ± 0.17 ^c^	6.80 ± 0.07	0.633 ± 0.034	8.70 ± 0.02
			6.62 ± 0.09	1.447 ± 0.022	8.92 ± 0.01
			6.42 ± 0.06	1.137 ± 0.021	8.99 ± 0.01

The EPS content (g/L) was based on total fermentation broth volume. FW = Fresh Weight; DW = Dry Weight; TPC = Total Plate Count at day 3; CFU = Colony-Forming Unit. Statistically significant differences among the values (*p* < 0.05) are indicated by different lowercase letters within each column.

**Table 4:** Antioxidant properties, levels of bioactive compounds, and antimicrobial activity of EPSs from RBR substrate by *Bacillus* spp. over 3 days.

Sample	Antioxidant Activities and Bioactive Contents					Antimicrobial Activity (Inhibition Zone in mm Diameter)	
	DPPH Radical Scavenging Activity (%)	FRAP (mg Fe^2+^/g DW)	Hydroxyl Radical Scavenging Activity (%)	TPC (mg GAE/g DW)	TFC (mg RE/g DW)	*S. agalactiae*	*S. aureus*
*B. amyloliquefaciens* KW1	63.38 ± 0.96 ^a^	5.14 ± 0.07 ^b^	58.52 ± 0.55 ^b^	15.71 ± 0.53 ^b^	25.43 ± 0.36 ^c^	8.02 ± 0.54 ^c^	7.33 ± 0.29 ^a^
*B. amyloliquefaciens* KW8	62.54 ± 0.62 ^a^	4.72 ± 0.04 ^c^	35.23 ± 0.25 ^c^	14.90 ± 0.25 ^b^	28.18 ± 0.19 ^b^	6.31 ± 0.27 ^e^	6.00 ± 0.50 ^b^
*B. amyloliquefaciens* KW9	51.91 ± 4.33 ^b^	3.43 ± 0.06 ^e^	22.81 ± 0.70 ^e^	15.49 ± 0.40 ^b^	22.72 ± 0.44 ^d^	8.33 ± 0.30 ^c^	5.10 ± 0.53 ^c^
*B. amyloliquefaciens* KW10	42.02 ± 1.70 ^c^	3.67 ± 0.11 ^e^	20.03 ± 1.67 ^f^	15.78 ± 0.34 ^b^	38.80 ± 0.44 ^b^	6.53 ± 0.19 ^e^	0.00 ± 0.00 ^d^
*B. tequilensis* PS21	65.50 ± 0.31 ^a^	6.51 ± 0.10 ^a^	80.53 ± 0.87 ^a^	17.98 ± 0.57 ^a^	39.14 ± 0.33 ^a^	10.07 ± 0.61 ^b^	7.83 ± 0.76 ^a^
*B. tequilensis* PS22	38.81 ± 1.04 ^d^	3.32 ± 0.20 ^e^	24.71 ± 0.92 ^d^	15.63 ± 0.76 ^b^	15.89 ± 0.13 ^g^	5.97 ± 0.84 ^f^	5.50 ± 0.50 ^c^
*B. tequilensis* PS23	34.89 ± 1.22 ^e^	4.22 ± 0.09 ^d^	23.38 ± 0.60 ^de^	15.36 ± 0.82 ^b^	18.22 ± 0.19 ^f^	11.28 ± 0.54 ^a^	6.33 ± 1.04 ^b^
*B. methylotrophicus* PS24	35.81 ± 0.38 ^f^	3.41 ± 0.06 ^e^	22.69 ± 0.56 ^e^	15.66 ± 0.64 ^b^	20.51 ± 0.54 ^e^	7.50 ± 0.50 ^d^	7.00 ± 0.87 ^a^

Statistical significance at *p* < 0.05 is indicated by different lowercase letters within the same column.

**Table 5:** Experimental runs based on Central Composite Design (CCD) with RBR, SBM, pH, and temperature as independent variables and EPS production and bacterial count as responses over 3-day fermentation using *B. tequilensis* PS21.

Run	Levels of Variation				Responses	
	X_1_ RBR (%)	X_2_ SBM (%)	X_3_ pH	X_4_ Temperature (°C)	Experimental EPS Production (g/L)	Experimental Bacterial Count (logCFU/mL)
1	1	−1	−1	−1	21.30	10.82
2	−1	−1	1	−1	21.30	10.15
3	0	2	0	0	38.50	12.86
4	−2	0	0	0	34.20	12.84
5	−1	−1	−1	−1	20.00	10.35
6	1	−1	1	−1	19.80	9.73
7	0	0	0	0	38.70	12.86
8	−1	1	−1	−1	21.00	10.48
9	1	1	1	1	30.00	10.48
10	1	1	−1	1	27.80	10.21
11	−1	−1	1	1	25.00	12.00
12	0	0	0	0	36.40	12.86
13	0	0	0	0	37.10	12.86
14	0	−2	0	0	22.30	10.90
15	0	0	0	0	38.50	12.68
16	0	0	−2	0	13.40	9.52
17	0	0	2	0	21.2	10.42
18	1	−1	1	1	21.8	12.19
19	−1	−1	−1	1	24.80	10.42
20	1	−1	−1	1	21.60	10.75
21	−1	1	1	−1	25.00	10.75
22	1	1	1	−1	21.50	10.48
23	1	1	1	−2	21.10	10.35
24	0	0	−1	−2	19.30	9.85
25	0	0	0	0	38.70	12.86
26	0	0	0	0	37.50	12.86

**Table 6:** Analysis of variance (ANOVA) for EPS production from RBR and SBM using *B. tequilensis* PS21 over 3 days.

Source	Sum of Squares	df	Mean Square	F Value	*p*-Value Prob > F	
Model	1503.85	14	107.42	37.21	<0.0001	significant
A—Carbon	43.09	1	43.09	14.93	0.0026	
B—Nitrogen	188.94	1	188.94	65.46	<0.0001	
C—pH	28.16	1	28.16	9.75	0.0097	
D—temp	109.28	1	109.28	37.86	<0.0001	
AB	10.09	1	10.09	3.5	0.0884	
AC	4.80	1	4.8	1.66	0.2236	
AD	18.89	1	18.89	6.55	0.0266	
BC	7.22	1	7.22	2.5	0.1421	
BD	48.45	1	48.45	16.78	0.0018	
CD	0.78	1	0.78	0.27	0.6131	
A^2^	67.96	1	67.96	23.54	0.0005	
B^2^	102.25	1	102.25	35.42	<0.0001	
C^2^	716.19	1	716.19	248.12	<0.0001	
D^2^	110.67	1	110.67	38.34	<0.0001	
Residual	31.75	11	2.89			
Lack of Fit	27.10	6	4.52	4.86	0.0518	not significant
Pure Error	4.65	5	0.93			
Cor Total	1535.60	25				
Std. Dev.	1.70		R-Squared	0.9793		
Mean	26.84		Adj R-Squared	0.9530		
C.V.%	6.33		Pred R-Squared	0.7840		
PRESS	331.65		Adeq Precision	18.1200		

**Table 7:** Optimal conditions for maximum EPS production from RBR and SBM using *B. tequilensis* PS21 over 3 days.

Condition	Carbon %	Nitrogen %	pH	Temperature (°C)	Desirability	Predicted EPS (g/L)	Actual EPS (g/L)	Mean Difference
1	5.19	3	6.69	38.76	0.977	40.14	39.05 ± 0.13	1.09
2	5.17	3	6.68	38.6	0.977	40.20	39.34 ± 0.12	0.86
3	5	3	6.66	39.51	0.974	41.17	39.82 ± 0.03	1.36
4	4.71	3	6.47	35.23	0.956	40.21	39.39 ± 0.13	0.82

**Table 8:** Cytotoxicity (%) and IC_50_ values of EPS extract on MCF-7 and HeLa cancer cells.

Treatment Time (h)	MCF-7		HeLa	
	Cytotoxicity (%)	IC50 (µg/mL)	Cytotoxicity (%)	IC50 (µg/mL)
24	55.61 ± 2.01 ^c^	1069.23 ± 146.11 ^b^	82.26 ± 2.91 ^b^	396.83 ± 13.23 ^c^
48	65.00 ± 1.79 ^b^	307.97 ± 7.57 ^b^	81.26 ± 0.63 ^b^	294.60 ± 4.91 ^b^
72	75.44 ± 1.58 ^a^	226.60 ± 7.17 ^a^	87.24 ± 0.83 ^a^	224.30 ± 10.06 ^a^

Superscripts denote statistical differences (*p* < 0.05) in the columns.

## Data Availability

The authors are unable to or have chosen not to specify which data has been used.

## Abbreviations

EPS—exopolysaccharide; RBR—riceberry broken rice; SBM—soybean meal; TSB—Tryptic Soy Broth; DNS—dinitrosalicylic acid; DPPH—2,2-diphenyl-1-picrylhydrazyl; FRAP—ferric reducing antioxidant power; TPC—total phenolic content; TFC—total flavonoid content; CFU—colony-forming unit; CCD—central composite design; RSM—response surface methodology; SEM—scanning electron microscopy; EDX—energy-dispersive X-ray spectroscopy; NMR—nuclear magnetic resonance; FTIR—Fourier-transform infrared spectroscopy; GPC—gel permeation chromatography; HPLC—high-performance liquid chromatography; XRD—X-ray diffraction; TGA—thermogravimetric analysis; DTG—derivative thermogravimetry; Mw—weight-averaged molecular weight; Mn—number-averaged molecular weight; MAS—magic angle spinning; DMEM—Dulbecco’s Modified Eagle Medium; FBS—fetal bovine serum; MTT—3-(4,5-dimethylthiazol-2-yl)-2,5 diphenyl tetrazolium bromide; DMSO—dimethyl sulfoxide; PBS—phosphate-buffered saline; OD—optical density; PE—porcine elastase; AAAPVN—N-Succinyl-Ala-Ala-Ala-p-nitroanilide.
